# Characterization of the complete mitochondrial genome of Oriental reed warbler, *Acrocephalus orientalis* (Aves: Passeriformes), and a comparative analysis with other *Sylviidae* species

**DOI:** 10.1080/23802359.2020.1756946

**Published:** 2020-05-12

**Authors:** Sung Hyun Kim, Soon Kyoo Choi, Seungki Lee

**Affiliations:** a Strategic Planning Division, National Institute of Biological Resources, Incheon, Republic of Korea; b Division of Forest Science, Kangwon National University, Chuncheon, Republic of Korea; c Biological and Genetic Resources Assessment Division, National Institute of Biological Resources, Incheon, Republic of Korea

**Keywords:** Mitochondrial genome, Passeriformes, *Sylviidae*, *Acrocephalus orientalis*

## Abstract

The complete mitochondrial genome of the Oriental reed warbler, *Acrocephalus orientalis*, which belongs to the family *Sylviidae* was determined. The complete mitochondrial genome has a length of 16,971 bp and consists of 13 protein-coding genes, 22 *tRNA* genes, 2 *rRNA* genes, and a control region. *A*. *orientalis* has a mitochondrial gene arrangement that is typical of vertebrates. Phylogenetic analysis using mitochondrial genomes of 11 related species revealed that *A*. *orientalis* is clustered with *Acrocephalus scirpaceus* and rooted with the other *Sylviidae* species. This mitochondrial genome provides an important resource for addressing taxonomic issues and studying molecular evolution.

The Oriental reed warbler, *Acrocephalus orientalis* (Passeriformes: *Sylviidae*), is a relatively large-bodied (18–20 cm in length) migratory bird native to East and Southeast Asia. This species occupies a unique ecological niche resulting from their long-range migration between breeding (China, Korea, and Japan) and winter habitats (Southeast Asia to Philippines and Indonesia). These traits also make them particularly vulnerable to habitat destruction and the effects of climate change. In this study, we determined the complete mitochondrial DNA sequence of *A*. *orientalis* and compared the sequence with those of other species of *Sylviidae* to analyze the phylogenetic relationship between them.

The *A*. *orientalis* specimen was collected from Heuksan Island, Republic of Korea (34.41N, 125.25E). Total genomic DNA was extracted from the specimen tissue, which has been deposited at the National Institute of Biological Resources (NIBR) (Voucher No. NIBRGR0000134238). The mitogenome was sequenced using Illumina Hiseq 4000 sequencing platform (Illumina, San Diego, CA) and assembled with *SOAPdenovo* at Macrogen Inc. (Seoul, Korea). The complete mitochondrial genome was annotated using MacClade version 4.08 (http://macclade.org/) (Maddison and Maddison 2005) and tRNAscan-SE version 2.0 (http://trna.ucsc.edu/tRNAscan-SE/) (Lowe and Chan 2016). All sampling activities were conducted in accordance with the Guidelines of Animal Ethics published by the NIBR.

The complete mitochondrial genome of *A*. *orientalis* (GenBank accession no. LC532223) is 16,971 bp in length and includes 13 protein-coding genes, 22 tRNA genes, 2 rRNA genes, and a control region. The overall base composition is 29.89% A, 31.97% C, 15.09% G, and 23.05% T. Similar to the mitogenomes of other vertebrates, the AT content is higher than the GC content (Saccone et al. 1999). The *12S rRNA* (1181 bp) and *16S rRNA* genes (1606 bp) are located between tRNA^Phe^ and tRNA^Val^ and between tRNA^Val^ and tRNA^Leu(UUR)^, respectively. The start codon of the 13 protein-coding genes begins with the sequence ATG. The stop codon of the protein-coding genes is TAA (*ND2, COII*, *ATP8*, *ATP6*, *ND3*, *ND4L*, *Cytb*, and *ND6*), TA (*ND1*), AGG (*COI*), T (*COIII* and *ND4*), and AGA (*ND5*). A control region (106 bp) is located between tRNA^Glu^ and tRNA^Phe^.

The phylogenetic trees were constructed by the maximum-likelihood method with 1000 replicates using MEGA version 7.0 software (MEGA, Philadelphia, PA) (Kumar et al. 2016). We compared the phylogenetic trees of the newly sequenced genome and 10 other complete *Sylviidae* species mitochondrial genome sequences acquired from the National Center for Biotechnology Information. We confirmed that *A*. *orientalis* is clustered with *Acrocephalus scirpaceus* (Singh et al. 2008) and rooted with the other Sylviidae species (Cai et al. 2019; Sun et al. 2020) (Figure 1). This mitochondrial genome provides an important resource for addressing taxonomic issues and studying molecular evolution.

**Figure 1.**
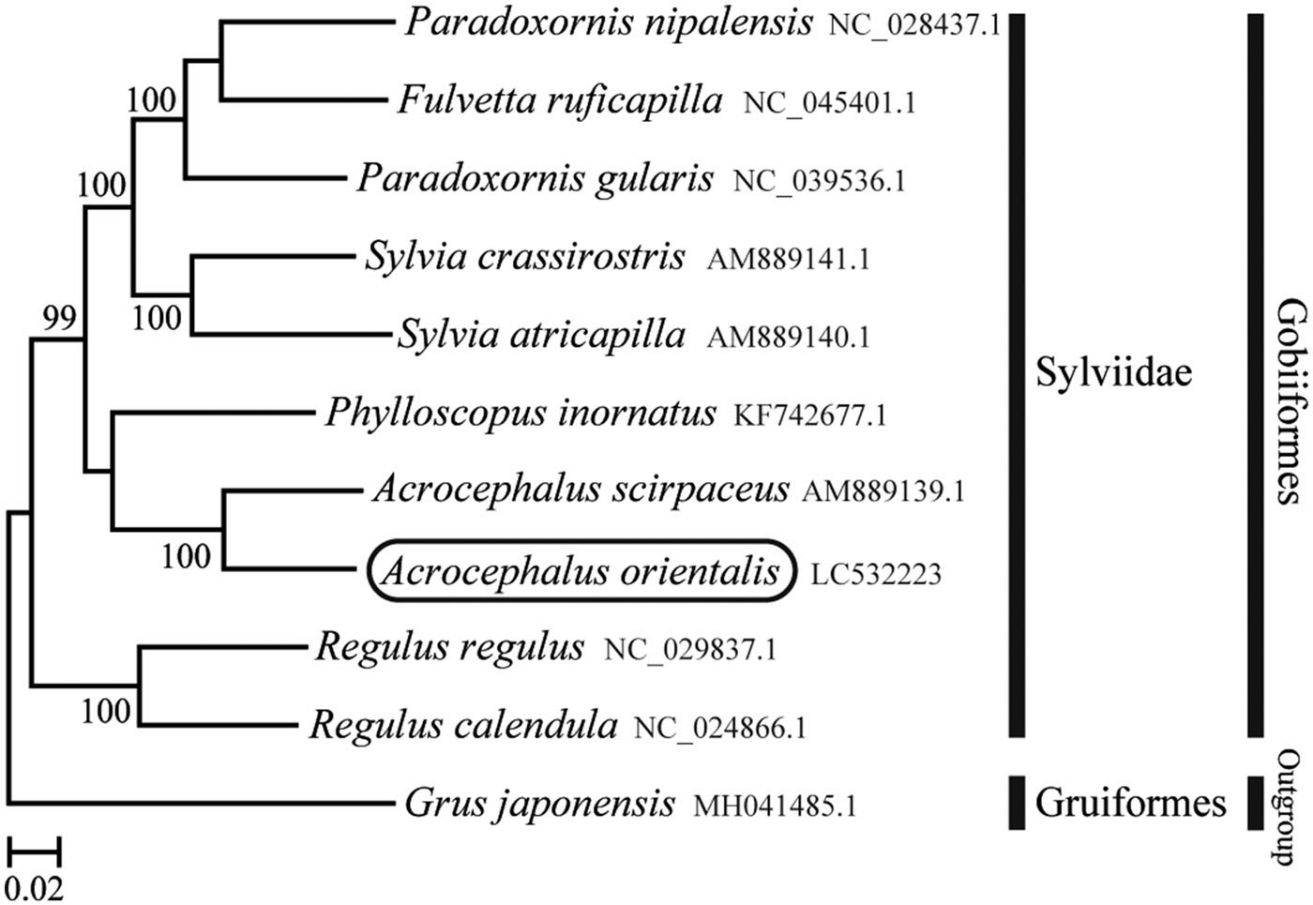
Phylogenetic position of *Acrocephalus orientalis* based on a comparison with the complete mitochondrial genome sequences of 10 other *Sylviidae* species. The analysis was performed using MEGA 7.0 software. The accession number for each species is indicated after the scientific name.

## Data Availability

The data that support the findings of this study are openly available in the DNA Data Bank of Japan (accession no. LC532223) at https://www.ddbj.nig.ac.jp.
